# Virtual Dermatological Congresses: A Chance for Equality and Diversity in Continuous Medical Education

**DOI:** 10.5826/dpc.1104a142

**Published:** 2021-10-01

**Authors:** Kerasia-Maria Plachouri, Francesk Mulita, Evangelia Kalloniati, Sophia Georgiou

**Affiliations:** 1 Dermatology Department, University General Hospital of Patras, Greece; 2 Department of Surgery, University General Hospital of Patras, Greece

Despite the unquestionable contribution of in-person dermatological congresses to knowledge sharing and networking, the associated health risk of large-scale social gatherings during the COVID-19 pandemic has affected the way we attend congresses, paving the way for digital, platform-based, online conferences [1].

Virtual dermatological congresses indeed offer a series of important advantages, such as flexibility in the attendance of multiple pre-recorded or live sessions, quick access to supplementary material, as well as a minimal ecological footprint since long-distance travelling or production of single-use congress material are suddenly out of the picture [2].

Another equally significant argument for the realization of virtual meetings, however, is the opportunity for physicians and academics who would normally be excluded from prestigious scientific meetings due to budgetary difficulties, to participate [3]. With very few exceptions, the majority of prominent national or international dermatological conferences involve registration fees that are usually beyond the financial means of participants that reside in low-income or developing countries [4]. Even in cases where registration fee waivers or scholarships are available, travel and accommodation costs can constitute a considerable obstacle to some, leading to inequality issues as far as educational opportunities are concerned [4]. Online congresses have no accommodation and transportation costs, and even registration fees tend to be reduced compared to in-person events. This represents a plus for financially weaker audiences [3,5]. Another important aspect is the fact that the shift of medical congresses towards virtual formats and the reduced costs, can give the chance to lower-income countries researchers or researchers with inadequate funding, to participate to these events, both as attendees and as presenters [6]. Thus, together with the enrichment of their educational agenda, underprivileged scientists have the chance to raise attention to their research, potentially paving the way for future funding or international collaborations [6].

After several months of social distancing and impaired educational and scientific activities, the dermatological community is eager to return to the “life as we know it” and the traditional in-person meetings with the obvious advantages that accompany them. The pandemic, however, has taught us that digitalization can also serve as a tool to promote equity and diversity among scientists and physicians. It is crucial to acknowledge this valuable lesson and to take appropriate action towards this direction even when this global challenge will be over. Examples of such initiatives constitute for instance, the organization of conferences with a dual format, including a virtual platform for audiences that are unable to attend due to unavailability of financial sponsorship.
